# Nano-optomechanical fiber-tip sensing

**DOI:** 10.1038/s44310-024-00011-y

**Published:** 2024-06-03

**Authors:** Arthur L. Hendriks, Luca Picelli, René P. J. van Veldhoven, Ewold Verhagen, Andrea Fiore

**Affiliations:** 1 Department of Applied Physics and Science Education, and Eindhoven Hendrik Casimir Institute, Eindhoven University of Technology, Eindhoven, The Netherlands; 2 Center for Nanophotonics, AMOLF, Amsterdam, The Netherlands

**Keywords:** Photonic crystals, Optical sensors, Optomechanics, Nanophotonics and plasmonics

## Abstract

Nano-optomechanical sensors exploit light confinement at the nanoscale to enable very precise measurements of displacement, force, acceleration, and mass. Their application is hampered by the complex optical set-ups or packaging schemes required to couple light to and from the nano-optomechanical resonator. In this work, we present a fiber-coupled nano-optomechanical sensor that requires no coupling optics. This is achieved by directly placing a nano-optomechanical structure, a double membrane photonic crystal (DM-PhC), on the facet of a fiber, using a simple and scalable wafer-to-fiber transfer method. The device is probed in reflection and has a resonance at telecom wavelengths with a relatively broad spectral width of 3–10 nm, which is advantageous for a simple read-out and achieves a displacement imprecision of $10\,{{\rm{fm}}}/{\sqrt{{\rm{Hz}}}}$. Using resonant driving and a ringdown measurement, we can induce and monitor mechanical oscillations with an nm-scale amplitude via the fiber, which allows for tracking the mechanical resonant frequency and the mechanical linewidth with imprecisions of 79 and 12 Hz, respectively, at integration times of 4.5 s. We further demonstrate the application of this fiber-tip sensor to the measurement of pressure, using the effect of collisional damping on the mechanical linewidth, leading to the imprecision of $9\times {10}^{-4}\,{\rm{mbar}}$ with an integration time of 290 s. This combination of optomechanics and fiber-tip sensing may open the way to a new generation of fiber sensors with unprecedented functionality, ultrasmall footprint, and low-cost readout.

## Introduction

Optomechanics studies the interaction between light and mechanical motion mediated by optical forces^1^. It is mostly based on systems where a mechanical deformation changes the properties of an optical resonance. The optomechanical interaction can be greatly enhanced by confining light in high-quality optical micro- or nanocavities, with examples such as microtoroids^2,3^, one- or two-dimensional photonic crystal cavities^4–10^. It can be used to read out the vibrations of low-mass mechanical resonators with extreme precision by measuring the optical spectrum, where displacement imprecisions at the ${{\rm{fm}}}/{\sqrt{{\rm{Hz}}}}$ level are routinely obtained, with values as low as ${10}^{-19}\,{{\rm{m}}}/{\sqrt{{\rm{Hz}}}}$ being reached in selected systems^11–14^. This allows for the sensing of physical quantities such as force, mass, pressure, and acceleration with ultimate precision^15–20^. Nevertheless, practical sensing applications of these nano-optomechanical structures are hindered by the complexity and the limited efficiency of light coupling to and from the sensor, related to the tight optical confinement and the narrow linewidths that are typically employed. Efforts have been made to couple chip-scale cavity-optomechanical devices to conventional optics such as fibers, where high fiber-to-cavity coupling efficiencies have been obtained by coupling adiabatic waveguide tapers to fibers^21,22^. Fiber-based optical cavities without active alignment have also been considered^23^. In this work, we show that precise mechanical measurements are possible using a nano-optomechanical structure placed directly on a fiber tip, with the radiation of the resonant mode matching the fiber mode and a relatively low optical quality factor. This configuration, enabled by an optimized optomechanical photonic crystal design and scalable membrane-on-fiber technology, enables large coupling efficiency without any external optics and with uncritical spectral alignment and provides displacement imprecisions down to $\frac{\delta {x}_{{{\rm {noise}}}}}{\sqrt{\Delta f}}=10\,{{\rm{fm}}}/{\sqrt{{\rm{Hz}}}}$. The fiber-tip optomechanical structure can readily be used in relevant sensing applications. Here, we discuss the possibility of measuring accreted mass by monitoring the mechanical frequency, and we experimentally demonstrate vacuum pressure sensing by monitoring the linewidth of the mechanical resonance using a ringdown approach.

## Results and discussion

### Design

**Fig. 1:**
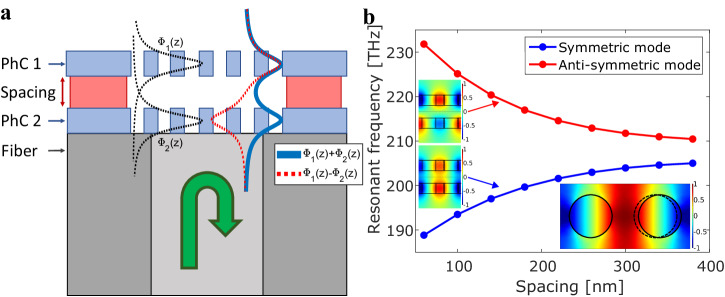
Design of nano-optomechanical fiber-tip sensor. **a** Side-view sketch of the device (not to scale), with the sketched profiles of the uncoupled modes (black) and of the supermodes (blue/red) of the two identical PhC membranes on the tip of a fiber. **b** Simulated resonance frequency of the optical supermodes as a function of the spacing between the membranes. The insets show the normalized electric field (*E*_*y*_) distributions of the modes for a single unit cell, where the second hole is shifted by 25 nm from its position in the original square lattice (dashed circle) to induce band folding.

The nano-optomechanical device used in this work is an InP double-membrane photonic crystal (DM-PhC) on the tip of a cleaved single-mode fiber (SMF-28: 9 μm core, 125 μm cladding, 0.14 numerical aperture) as sketched in Fig. 1a. Here, the evanescent fields of the two PhC modes (in black) overlap and form a coupled system with two modes: the symmetric (in blue) and anti-symmetric (in red)^9,24–26^. The photonic crystals have a rectangular unit cell where every second hole is shifted by a small amount in the *x*-direction to induce band folding^27–29^. This produces an extra loss channel at the $\Gamma$-point, resulting in a significant vertical coupling of the DM-PhC with the fiber mode. The optical and mechanical response can then be read out in reflection with a fiber-coupled setup. Using finite-element method (FEM: COMSOL Multiphysics^30^) simulations, two guided-mode resonances are found, as seen in Fig. 1b (see the “Methods” section). The resonant frequencies of both modes are affected by the separation between the two PhC membranes. The optomechanical coupling strength can be derived from the optical resonance frequency shift per unit of displacement, which, for the symmetric mode at the spacing used (220 nm), is approximately ${G}_{\omega }=2\pi \cdot \frac{\partial \nu }{\partial x}=2\pi \cdot 40\,{\rm{GHz}}/{\rm{nm}}$. This large coupling strength allows for optical measurements of small vertical displacements of the suspended top membrane.

### Fabrication

**Fig. 2:**
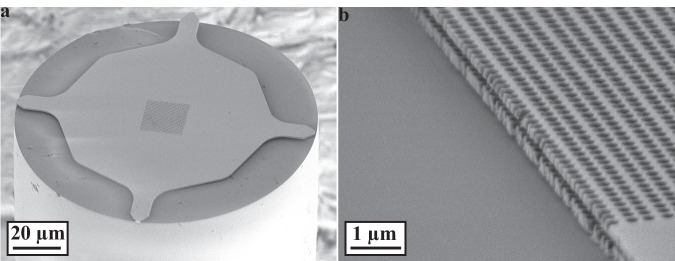
SEM images of a DM-PhC on a fiber. **a** SEM image of a DM-PhC on top of a fiber. **b** SEM image of a cleaved DM-PhC transferred to a fiber, where the separation between the two PhC membranes can clearly be seen.

The DM-PhCs were fabricated on two 180 nm InP membranes spaced by a 220 nm InGaAs membrane using standard semiconductor nanofabrication techniques and subsequently transferred to the tip of an optical fiber using the procedure presented in ref. ^31^ (see the “Methods” section). A scanning electron microscope (SEM) image of the DM-PhC on a fiber-tip can be seen in Fig. 2a. In Fig. 2b, an SEM image of another DM-PhC on a fiber-tip that has been cleaved before the transfer can be seen. Here, the two PhCs show a clear intermembrane separation confirming that optomechanical structures can be transferred with the aforementioned transfer technique. Additionally, Fig. 2b shows that the DM-PhC has minimal bending after a successful transfer. The suspended area of the DM-PhC is estimated to be around $\left(20\times 20\right)\,{\rm{\mu }}{{\rm{m}}}^{2}$ from the used etching time and Fig. 2b.

### Optical and thermomechanical noise measurements

**Fig. 3:**
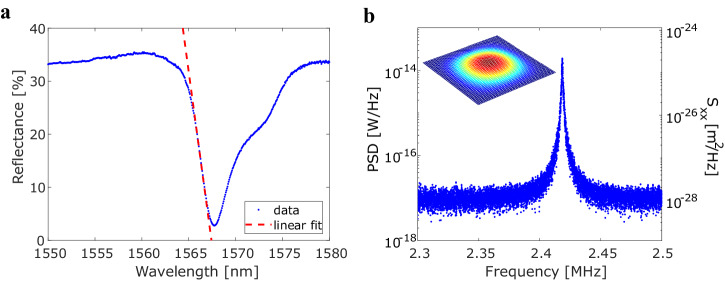
Optical and mechanical spectra of the nano-optomechanical fiber-tip sensor. **a** Optical resonance of the DM-PhC showing the absolute reflectance as a function of wavelength. A linear fit of the slope of the Fano resonance gives a maximum slope of 13%/nm. **b** Thermomechanical noise spectrum obtained by measuring the reflected laser power at the steepest slope of the resonance with an ESA. The inset shows a FEM simulation of the mechanical resonant mode of the top PhC membrane (see the “Methods” section).

The optical response of the DM-PhC on fiber can be seen in Fig. 3a. This DM-PhC has a lattice constant $a=368\,{\rm{nm}}$, radius $r=0.3a$, and shift $s=0.07a$. The value of the radius and shift were chosen in order to have an optimized trade-off between reflection modulation and *Q*-factor, similar to ref. ^31^ The reflectance spectrum is characterized by a fiber-coupled setup by illuminating the DM-PhC with a superluminescent diode (SLED) and measuring the reflected spectrum with an optical spectrum analyzer (OSA). For this measurement, a manual fiber polarization controller is used to maximize the visibility of the resonance. The reflectance is measured by dividing the acquired spectrum by a reference spectrum which is obtained by using a fiber-coupled retroreflector. The spectrum has an asymmetric Fano line shape with a dip around 1567 nm and a quality factor of 330. The lineshape corresponds to the simulations. However, the resonance wavelength is lower than simulated, which is most likely due to an over-etch in the fabricated devices. Moreover, the quality factor is lower for the fabricated devices compared to the simulations (*Q* = 800), which is most likely due to the larger fabricated hole size and the conicity (asymmetry in hole size of the top and bottom slab). The *Q*-factor is lower compared to previous demonstrations of DM-PhCs on chip^9^ since, in this work, a PhC with a guided-mode resonance is used^24^ and not a photonic crystal cavity which will couple poorly with the fiber mode. The guided-mode resonance of the DM-PhC couples efficiently with the fiber mode, which results in large visibility (absolute reflectance modulation $\Delta R=32.7 \%$) and, therefore, also a steep slope of maximally $\frac{\Delta R}{\Delta \lambda }=13 \% /{\rm{nm}}$. The optical resonance wavelength shift due to a change in the intermembrane separation can be tracked by placing a near-infrared tunable laser (Santec TSL-710) at the wavelength corresponding to the steepest slope and measuring the reflected power using a fixed-gain amplified photodetector (Thorlabs PDA10CF-EC). The signal is analyzed in the frequency domain using an electronic spectrum analyzer (ESA: Rhode & Schwarz FSV3004). At low enough pressures a Lorentzian peak is measured for each mechanical mode, associated with random thermally driven motion, which is referred to as thermomechanical noise^1^. The thermomechanical noise spectrum of the device is depicted in Fig. 3b, showing a mechanical frequency of ${\Omega }_{{\rm{m}},\exp }/2\pi =2.42\,{\rm{MHz}}$ and a mechanical linewidth of ${\Gamma }_{{\rm{m}}}/2\pi =1.1\,{\rm{kHz}}$. The inset of Fig. 3b shows the fundamental flexural mode obtained by FEM, which has a calculated mechanical frequency at ${\Omega }_{{\rm{m}},{\rm{sim}}}/2\pi =2.53\,{\rm{MHz}}$ and an effective mass ${m}_{{\rm{eff}}}=70\,{\rm{pg}}$ (see the “Methods” section). The power spectral density (PSD) of Fig. 3b is proportional to the displacement spectral density (${S}_{{xx}}$) through multiple parameters of the transduction chain. In order to circumvent the uncertainty in their determination, we calibrate the ${S}_{{xx}}$ axis in Fig. 3b by imposing the equipartition theorem:

1 $$\frac{1}{2}{m}_{{\rm{eff}}}{\Omega }_{{\rm{m}}}^{2}{\left\langle {x}^{2}\right\rangle }_{{\rm{th}}}=\frac{1}{2}{k}_{{\rm{B}}}T$$

where ${m}_{{\rm{eff}}}$ is the effective mass found from FEM simulations, $T$ is the temperature, and the mean square of the displacement fluctuations is given as^1^

2 $${\left\langle {x}^{2}\right\rangle }_{{\rm{th}}}={\int }_{-\infty }^{+\infty }{S}_{{xx}}\left(\Omega \right)\frac{d\Omega }{2\pi }$$

The noise floor of ${S}_{{xx}}$ is equal to the displacement imprecision, which in this case is $\frac{\delta {x}_{{\rm{noise}}}}{\sqrt{\Delta f}}=10\,{{\rm{fm}}}/{\sqrt{{\rm{Hz}}}}$ at an optical power of ${P}_{{in}}=250\,{\rm{\mu }}{\rm{W}}$ and is limited by electronic noise. This could be improved through homodyne readout. The displacement imprecision in this work is an order of magnitude lower compared to previously demonstrated DM-PhCs^9^, but higher than the most precise nano-optomechanical sensors^14,16^. We stress that with this approach, differently from these previous works, our sensor does not require active optical alignment and coupling optics, and given the very small dimension of the fiber it is suitable for integration within production systems. Additionally, the relatively large resonance linewidth makes it possible to use a fixed-wavelength laser for the interrogation, releasing the need for a tunable laser.

### Optical driving

**Fig. 4:**
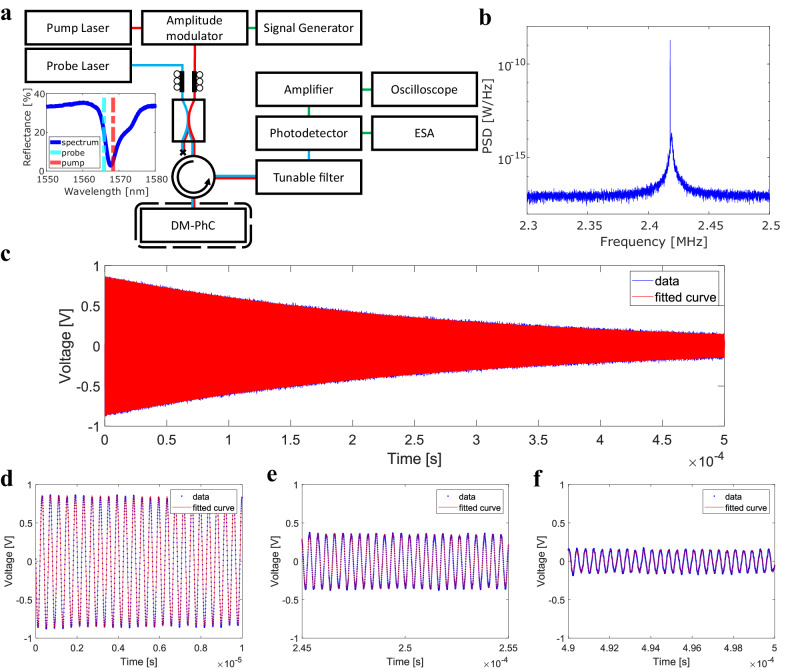
Setup for the ringdown measurements. **a** Setup used for the driven measurements. An amplitude-modulated pump laser (red line) and a probe laser (cyan line) are combined and coupled to the sensor. Before the photodetector, the pump laser is filtered out so only the probe laser is measured. The response is read-out using an ESA or an oscilloscope (electronic connections in green). **b** Power spectral density of the photocurrent generated by the reflected probe laser in the presence of a modulated pump laser. **c** Ringdown of DM-PhC after the pump laser is switched off. From the fit, the resonant frequency and the linewidth are obtained. **d–f** Zoom of the ringdown on three different time windows.

The frequency and linewidth of the mechanical mode will change upon a change in the mass (e.g. due to mass accretion on the surface) or a change in the ambient vacuum pressure. This allows for the application of the fiber-tip DM-PhC as a sensor for such parameters. The frequency and the linewidth of the resonator should therefore be measured with high precision, which can be done by increasing the amplitude of the mechanical oscillation and, therefore, the signal-to-noise ratio^32^. To this aim, we resonantly actuate the top membrane by employing a pump-probe configuration where the optomechanical gradient force of a modulated pump laser brings the DM-PhC into motion. The setup used for this measurement is shown in Fig. 4a. Here, an interrogation laser (probe laser: Santec TSL-710) is combined with a pump laser (ID Photonics CBDX1), which is amplitude-modulated by an electro-optic modulator (EOM: iXblue MXAN-LN-10) with a frequency and amplitude set by a function generator (HP 33120A). After reflection from the sample, the light from the pump laser is filtered out by a fiber-coupled optical tunable filter (Santec OTF-300), so only the power from the probe laser is detected by the photodetector. In this configuration, the wavelength of the probe laser is tuned to the steepest slope of the resonance ($1565.9\,{\rm{nm}}$), and the pump laser wavelength is detuned to the other side of the resonance ($1568.4\,{\rm{nm}}$) in order to be completely out of the bandwidth of the tunable filter. The frequency of the function generator is swept over the natural oscillation frequency, and the mechanical peak is read out using the ESA. An example of a driven spectrum is seen in Fig. 4b, where the frequency of the pump is set to 2.418 MHz, producing a coherent oscillation at this frequency (sharp peak in the spectrum, not resolved due to the limited resolution bandwidth of the ESA) on top of the thermomechanical noise. The coherent drive peak has an area that is 3 orders of magnitude larger than the integrated thermomechanical noise. The measurement of frequency and linewidth consists of a ringdown experiment, where the function generator is set to burst mode in order to modulate the pump laser and drive the mechanical resonance for ~0.41 ms, corresponding to 1000 mechanical cycles. When the pump laser modulation is switched off, the reflection of the probe laser is measured by the photodetector, which is subsequently amplified by a 60 dB voltage amplifier (Femto HVA-10M-60-B), and then displayed as a function of time using a digital oscilloscope (Siglent SDS2104X Plus). This measurement sequence takes 1 ms, and it is repeated multiple times (in this work, 32 averages are typically employed) to average out the corresponding time traces. A ringdown using 32 averages can be seen in Fig. 4c The ringdown is fitted with an exponentially decaying oscillation of the form:

3 $$V={V}_{0}\sin \left({\Omega }_{{\rm{m}}}\,t+\phi \right)\,\exp \left(-\frac{{\Omega }_{{\rm{m}}}\,t}{2{Q}_{{\rm{m}}}}\right)$$

where ${V}_{0}$ is the voltage amplitude, $t$ is the time, $\phi$ is the phase, and ${Q}_{\rm{{m}}}$ the mechanical *Q*-factor. Three magnified areas of the ringdown of Fig. 4c can be seen in Fig. 4d–f. From the fit, both the mechanical frequency and the mechanical linewidth (${\Gamma }_{{\rm{m}}}={\Omega }_{{\rm{m}}}/{Q}_{{\rm{m}}}$) are retrieved.

**Fig. 5:**
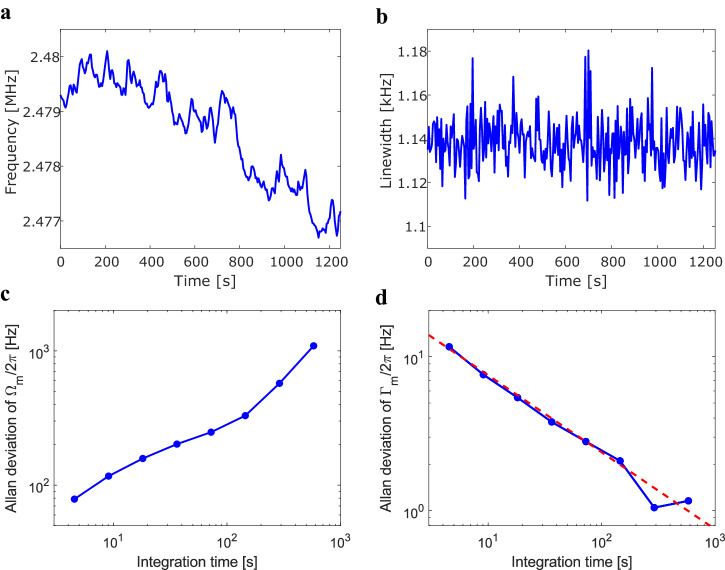
Frequency and linewidth stability measurements. **a** Fitted frequency over time, **b** Fitted linewidth over time. **c** Allan deviation of the frequency seen in (**a**); **d** Allan deviation of the linewidth seen in (**b**). The dashed red line indicates a ${{\rm{\tau }}}^{-\frac{1}{2}}$. scaling.

In order to find the imprecision in the frequency and linewidth, an Allan deviation analysis is performed, where the deviation $\sigma$ as a function of integration time $\tau$ is investigated. In Fig. 5a and b the stability over 20 min for the frequency and linewidth respectively are shown. The respective Allan deviation plots can be seen in Fig. 5c for the frequency and Fig. 5d for the linewidth. From Fig. 5a and c it can be seen that the frequency can be determined accurately at short time scales but has substantial drift over time. This is most likely due to small particles adsorbing or desorbing from the surface of the membrane, minor temperature fluctuations, or laser instability. From Fig. 5b and d it can be seen that the linewidth is stable over longer time scales. The dashed red line in Fig. 5d with a slope of ${\tau }^{-1/2}$ indicates that the precision of the system is dominated by white noise in the considered range of integration times^33^. At the shortest integration time (4.5 s) the imprecision in the frequency and linewidth are ${\sigma }_{\Omega ,\tau\, =\,4.5\,{\rm{s}}}/2\pi =78.8\,{\rm{Hz}}$ and ${\sigma }_{\Gamma ,\tau\, =\,4.5\,{\rm{s}}}/2\pi =11.6\,{\rm{Hz}}$, respectively. By integrating over a longer time, the imprecision in the linewidth can be improved to ${\sigma }_{\Gamma ,\tau\, =\,290\,{\rm{s}}}/2\pi =1.05\,{\rm{Hz}}$.

### Mass imprecision

The DM-PhC can be approximated as a simple harmonic oscillator with a mechanical frequency ${\Omega }_{{\rm{m}}}=\sqrt{\frac{k}{{m}_{{\rm{eff}}}}}$, where $k$ is the spring constant. Any mass variation resulting from e.g. accreted mass, produces a resonant frequency shift ${\delta \Omega }_{\rm{{m}}}$. Assuming that the mass variation is much smaller than the effective vibratory mass, $\delta{\rm{m}}\ll {m}_{{\rm{eff}}}$, the mass imprecision $\delta{\rm{m}}$ can be determined from the frequency imprecision ${\delta \Omega }_{\rm{{m}}}$ from:

3 $$\delta{\rm{m}}=2{m}_{{\rm{eff}}}\frac{{\delta \Omega }_{{\rm{m}}}}{{\Omega }_{{\rm{m}}}}$$

Utilizing Eq. 3 and the minimum imprecision in the frequency of Fig. 5c we obtain a mass imprecision of $\delta{\rm{m}}=4\,{\rm{fg}}$ when using an integration time of 4.5 s. This corresponds to a single polystyrene (${\rho }_{\rm{PS}}=1\,{\rm{g}}/{\rm{c}}{\rm{m}}^{3}$) particle with a diameter of 200 nm or a single gold particle (${\rho }_{\rm{Au}}=19.3\,{\rm{g}}/{\rm{c}}{\rm{m}}^{3}$) with a diameter of 75 nm. The mass sensitivity could be further enhanced by reducing the effective mass of the mechanical mode, e.g. using a GHz-frequency breathing mode^34^. In view of its relatively large area, the DM-PhC resonator can be better utilized as a precise sensor of deposited layer thickness, e.g. in deposition or epitaxy machines. By combining the mass imprecision with the effective area of the mechanical mode, the imprecision in the measurement of the thickness of a deposited layer can be estimated. For a thin layer of silicon dioxide $({\rho }_{\rm{{{SiO}}}_{2}}=2.65\,{\rm{g}}/{\rm{c}}{{\rm{m}}}^{3})$ this would correspond to a thickness of $\delta{\rm{t}}=3\,{\rm{pm}}$, which is well below a monolayer thickness.

### Pressure sensing

**Fig. 6:**
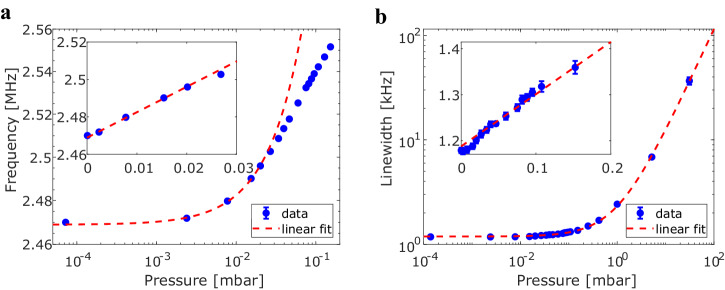
Frequency and linewidth dependence as a function of pressure. **a** Variation of mechanical frequency as a function of pressure showing a sensitivity of ${{\rm{S}}}_{{\rm{P}},\Omega }/2{\rm{\pi }}=1.36\,{\rm{MHz}}/{\rm{mbar}}$. Inset: zoom-in on the linear region. **b** Variation of mechanical linewidth as a function of pressure showing a sensitivity of ${{\rm{S}}}_{{\rm{P}},\Gamma }/2{\rm{\pi }}=1.14\,{\rm{kHz}}/{\rm{mbar}}$. Inset: Zoom in on the lower pressure range showing the linear relationship.

Pressure affects the mechanical peak of the DM-PhC in multiple ways. Firstly, the squeeze film effect effectively increases the spring constant and, therefore, the mechanical frequency when the pressure $P$ is increased^35^. This effect is seen in structures where gas is compressed in a thin layer below a vibrating membrane, which increases the stiffness depending on the gas pressure^36^. This pressure dependence of the mechanical frequency was previously used to demonstrate fiber-based optomechanical pressure sensing^37^. A sweep from vacuum to atmospheric pressure while measuring the mechanical frequency by ringdown is performed to obtain the pressure sensitivity for our DM PhC structure. This is plotted in Fig. 6a where the mechanical frequency ${\Omega }_{\rm{{m}}}/2\pi$ is plotted and a linear region is fitted in the ${10}^{-4}-2\times {10}^{-2}\,{\rm{mbar}}$ range with a sensitivity of ${S}_{{\rm{P}},\Omega }/2\pi =1.36\,{\rm{MHz}}/{\rm{mbar}}$. At higher pressures the dependence becomes nonlinear due to the squeeze film effect as described in ref. ^38^. Combining the sensitivity with the imprecision in the frequency results in an imprecision in the pressure of ${\sigma }_{{\rm{P}},\Omega }={\sigma }_{{\rm{f}}}/{S}_{{\rm{P}},\Omega }=6\times {10}^{-5}\,{\rm{mbar}}$. However, measuring the frequency to obtain the pressure has the drawback that the mechanical frequency continuously shifts (as seen in Fig. 5c) due to the accretion of mass. Indeed, it is observed that the frequency shifts by at least 2 kHz over the timespan of a day (SI). This makes the mechanical frequency an unsuitable parameter to determine the pressure for a practical sensor.

Here we propose using an alternative mechanism for determining the pressure, namely the kinetic damping due to atoms or molecules colliding with the resonator, where the friction force scales proportionally to the pressure^35^. This is also observed in the DM-PhC, as seen in Fig. 6b, where the linewidth of the mechanical resonance$\,{\Gamma }_{\rm{{m}}}/2\pi$ is plotted as a function of pressure. The relation between linewidth and pressure is linear in the ${10}^{-2}-{10}^{3}\,{\rm{mbar}}$ range with a sensitivity of ${S}_{{\rm{P}},\Gamma }/2\pi =1.14\,{\rm{kHz}}/{\rm{mbar}}$. The upper limit of the pressure range is due to a decrease in the amplitude of the oscillations, which makes the ringdown method unsuitable for the pressures above $50\,{\rm{mbar}}$. From measurements of the thermomechanical noise spectrum, we have observed that the linear dependence of linewidth on pressure continues up to atmospheric pressures, indicating the possibility of extending the range using a combination of measurement methods (SI). Combining the minimum imprecision in the linewidth (found at an integration time of $\tau =290\,{\rm{{s}}}$) and the sensitivity, a minimum imprecision in the pressure of ${\sigma }_{{\rm{P}},\Gamma }={\sigma }_{\Gamma }/{S}_{{\rm{P}},\Gamma }=9\times {10}^{-4}\,{\rm{mbar}}$ is obtained. A real-time measurement of the linewidth when increasing the pressure in small steps confirmed that pressure changes in the low ${10}^{-3}\,{\rm{mbar}}$ range can be measured with integration times of a few tens of seconds (SI). While this imprecision is higher than the one obtained from the pressure-dependence of the mechanical frequency^37^, as mentioned above the linewidth will result in a much more reliable pressure determination as compared to the frequency.

In conclusion, we demonstrated a simple, inexpensive, and scalable approach to the application of nano-optomechanical structures in fiber sensing, estimated their mass sensing capabilities, and demonstrated their application in vacuum pressure sensing. For mass sensing, we estimate a mass imprecision of $\delta{\rm{m}}=4\,{\rm{fg}}$, or equivalently an imprecision in the thickness of a deposited layer of $\delta{\rm{t}}=3\,{\rm{pm}}$. We determined the pressure by using the linewidth of the mechanical resonance in the range of ${10}^{-2}-{10}^{2}\,{\rm{mbar}}$ with an imprecision of ${\sigma }_{{\rm{P}},\Gamma }=9\times {10}^{-4}\,{\rm{mbar}}$ using a ringdown approach. The use of the mechanical linewidth provides a more reliable pressure measurement than the frequency, as the accretion of small masses shifts the mechanical frequency considerably.

## Methods

### Fabrication and assembly

The DM-PhC is fabricated using standard semiconductor growth, lithography, and etching techniques. Using metalorganic vapor-phase epitaxy (MOVPE), a 300 nm-thick lattice-matched In_0.53_Ga_0.47_As sacrificial layer is grown on top of a [100] InP substrate followed by two 180 nm-thick InP membranes spaced by a 220 nm-thick In_0.53_Ga_0.47_As layer. Afterward, a 200 nm-thick Si_3_N_4_ layer is deposited on top of the wafer using plasma-enhanced chemical vapor deposition (PECVD) as a hard mask. Electron beam lithography (EBL) is used to pattern both the PhCs and the surrounding support structures. Then, the pattern is transferred into the hard mask by reactive ion etching (RIE) with a CHF_3_ plasma, which is followed by etching through the two InP membranes and the InGaAs spacer layer using inductively-coupled-plasma reactive ion etching (ICP-RIE) with a CH_4_/H_2_ chemistry at 60 °C. Subsequently, the hard mask is removed by buffered hydrofluoric acid (BHF) and replaced by a 250 nm-thick Si_3_N_4_ protection layer. A 400 nm-thick Si_3_N_4_ hard mask is then deposited on the substrate side using PECVD for backside processing. Afterward, a second EBL is performed to pattern large windows on the substrate side, which are aligned with respect to the pattern on the device layer. This is then followed by dry etching of the Si_3_N_4_, wet etching of the InP substrate by a HCl:H_3_PO_4_ solution, removal of the Si_3_N_4_ by BHF, and etching of the sacrificial layer by H_2_O:H_2_SO_4_:H_2_O_2_. In this step also the InGaAs spacer between the two InP membranes is etched through the PhC holes, which suspends the top membrane with an area of $\left(20\times 20\right)\,{\rm{\mu }}{{\rm{m}}}^{2}$. Finally, the sample is dried using critical point drying (CPD) so that the suspended membranes do not collapse due to capillary forces.

The transfer to the fiber facet is achieved using a simple and scalable transfer technique that has previously been demonstrated in our group^31,39,40^. This process is similar to our previous work, however, this time, it is performed on a nano-optomechanical structure, where the proximity between the two suspended InP membranes makes the transfer more critical. The method relies on a support structure surrounding the optical device, which has indents positioned in correspondence with the circumference of an SM optical fiber (125 μm), acting as breaking points (see Fig. 2a). For this structure, the indents are slightly wider (2.5 μm instead of 1.75 μm) compared to their single-membrane counterparts to stop the structure from breaking during the final fabrication steps due to the thicker layer stack (580 nm compared to 250 nm). The large windows etched on the backside allow for the insertion of fiber through the substrate, which can then come into contact with the optomechanical structure. During the transfer, the fiber core is aligned with the center of the optomechanical structure using a microscope, a translation stage for the fiber, and a translation stage for the wafer. Upon contact, the tethers connecting the optomechanical device with the rest of the chip break and the DM-PhC is attached to the fiber facet without the use of any adhesives. An SEM image after the transfer of the DM-PhC to the fiber can be seen in Fig. 2a. A cleaved DM-PhC transferred to a fiber is shown in Fig. 2b. Here, it is clearly seen that the two membranes, spaced by only a few hundred nanometers, can be transferred to a fiber facet. The possibility of reproducibly transferring suspended optomechanical structures is a unique feature of the employed transfer method, setting it apart from other methods involving e.g. transfer printing^41,42^, liquid-assisted transfer of patterned membranes^43^, and manual transfer with a micromanipulator tip^44–47^.

### Finite element simulations

To simulate the DM-PhCs, the commercially available finite element method (FEM) simulation software COMSOL® was used^30^.

### Optical simulations

An overview of the optical simulations can be seen in Fig. S1 in the supplementary information (SI). The simulations were performed for an infinite DM-PhC by simulating one unit cell and utilizing periodic boundary conditions (PBC). Perfectly matched layers (PML) at the top and bottom boundaries were used to prevent reflections. The parameters used for the simulations are the same as for the measured DM-PhC. Eigenfrequency studies were performed to obtain the optical frequencies of the (anti-)symmetric modes. The simulations from Fig. 1b were performed by sweeping the value of the spacing layer between the two membranes.

### Mechanical simulations

An overview of the mechanical simulations can be seen in Fig. S2. The simulations were performed for a finite DM-PhC with $50\times 50$ holes, which is the same as the experimental structure. One free boundary is selected with an area of $\left(20\times 20\right)\,{\rm{\mu }}{{\rm{m}}}^{2}$, which is estimated from the etching time of fabricated samples, while the rest of the boundaries have a fixed constraint. An eigenfrequency is computed with a frequency at ${\Omega }_{{\rm{m}},{\rm{sim}}}/2\pi =2.53\,{\rm{MHz}}$ for the fundamental flexural mode (inset of Fig. 3b).

## Supplementary information

Supplementary Information

## Data Availability

The datasets used and/or analyzed during the current study are available from the corresponding author upon reasonable request.
