# Supplementary material for: Nano-optomechanical fiber-tip sensing
Source: Npj Nanophoton. 2024 Jun 3;1(1):10. doi: 10.1038/s44310-024-00011-y (PMC11879848; doi:10.1038/s44310-024-00011-y)
Supplement: Supplementary file 1 — Supplementary Information [file 44310_2024_11_MOESM1_ESM.pdf]

Nano-Optomechanical Fiber-Tip Sensing

Supplementary information

Arthur L. Hendriks[1,*]
Luca Picelli[1]
René P.J. van Veldhoven[1]
Ewold Verhagen[1,2]
Andrea Fiore[1]

[1] Department of Applied Physics and Science Education, and Eindhoven Hendrik Casimir Institute, Eindhoven University of Technology, Eindhoven, The Netherlands
[2] Center for Nanophotonics, AMOLF, Amsterdam, The Netherlands
[*] corresponding author: a.l.hendriks@tue.nl

FEM simulations

Optical simulations:

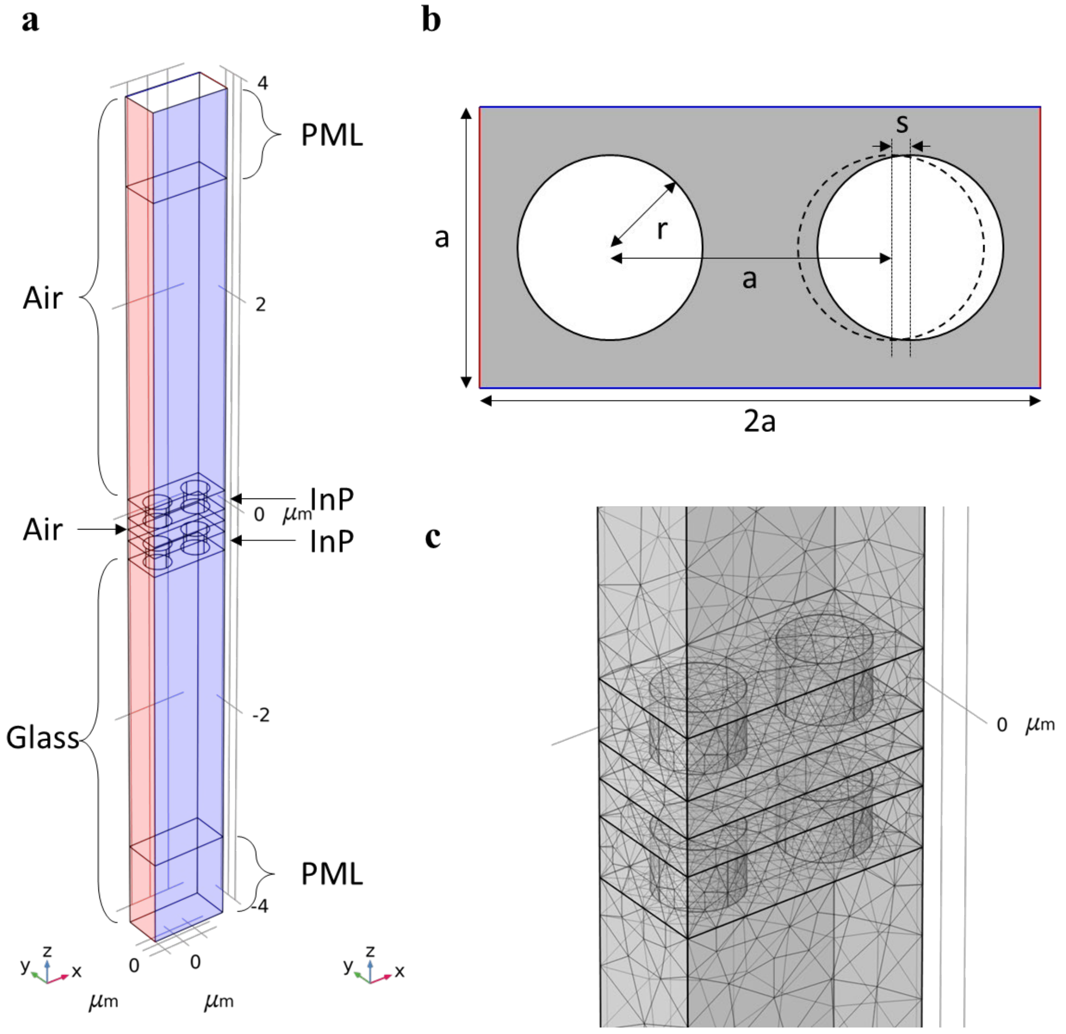

***Fig. S1: a)** Overview of the optical DM-PhC FEM simulations. One unit cell is simulated with periodic boundary conditions (PBC) indicated by the blue and red lines/surfaces which are applied on opposite sides. Perfectly matched layer (PML) boundary conditions are applied at the top and bottom in order to prevent reflections. **b)** Cut-plane through one of the membranes showing the definitions of the lattice constant (a), radius (r), and the shift (s). **c)** Zoom-in of the DM-PhC simulation domain showing the used meshing.*

Mechanical simulation:

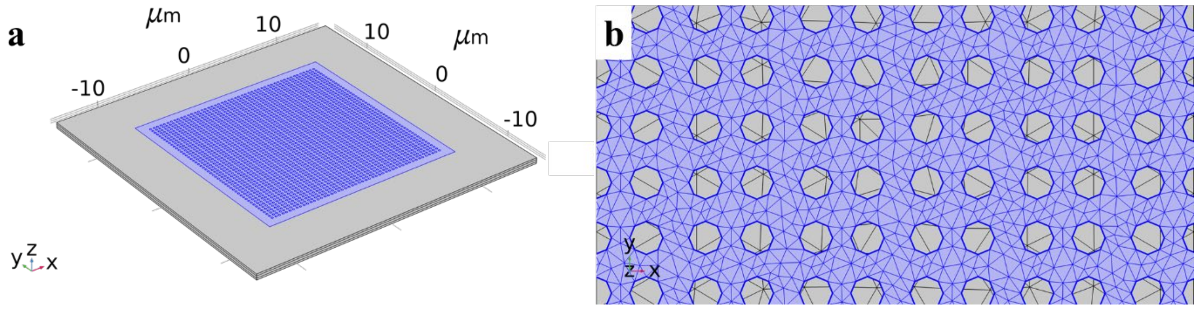

Fig. S2: a) *Overview of the mechanical DM-PhC FEM simulations. A suspended membrane with a* 50×50 *lattice of holes is simulated, where an area of* $(20 \times 20)\ \mu m^2$ *is used as a free boundary (indicated in blue), while the rest is fixed.* ***b)*** *Top view of the DM-PhC zoomed in on the holes showing the used meshing.*

Pressure dependence of linewidth at higher pressures

The ringdown method is unsuitable at pressures higher than $50\ \mathrm{mbar}$ due to a decrease in amplitude of the oscillation. However, the linear dependence of the linewidth of the DM-PhC continues up to atmospheric pressures. This was confirmed by measuring the linewidth of the thermomechanical noise spectrum at higher pressures using the ESA as seen in Fig. S3.

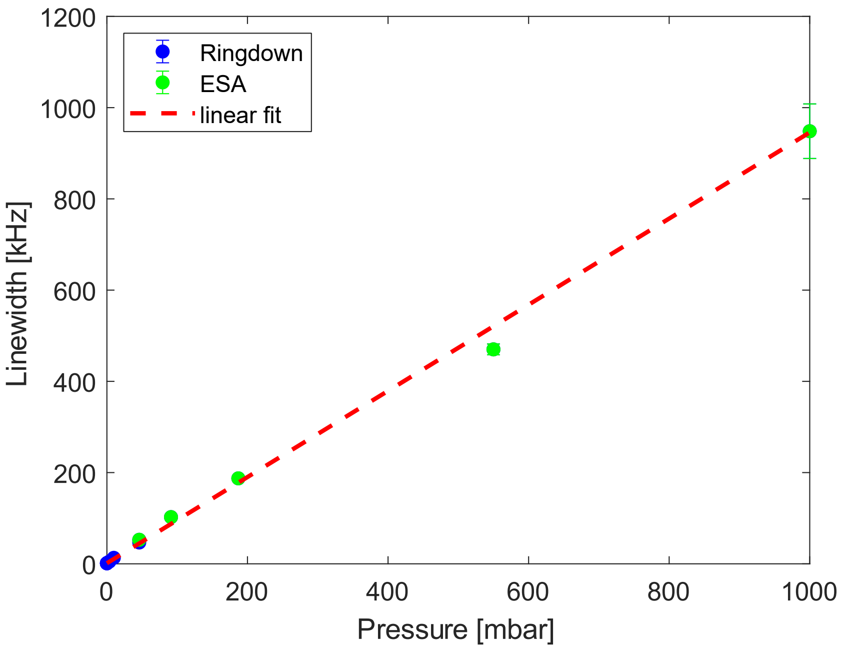

Fig. S3: *Linear dependence of linewidth of DM-PhC up to atmospheric pressures. The blue dots correspond to measurements performed with the ringdown method and the green dots to measurement with the ESA.*

Mechanical frequency drift over time

The mechanical frequency drifts on relatively short timescales as seen in Fig. 5a. Additionally, on longer timescales, in the order of days, it also drifts with several tens of kHz as seen in Fig. S4a. Here, 50 ringdowns (where a ringdown is an average of 32 oscilloscope traces) were performed per data point which were subsequently fitted to find the frequency and the linewidth. The measurement was repeated over a span of four days. As can be seen the mechanical linewidth remains relatively constant over the span of multiple days, while the frequency drifts substantially. This makes the linewidth a more suitable parameter to track the pressure compared to the frequency.

Experimental imprecision in pressure

In the main text we predict the imprecision of the sensor to pressure ($\sigma_{P,\Gamma}$) by combining the values of the imprecision in the linewidth ($\sigma_{\Gamma}/2\pi$) and the sensitivity ($S_{P,\Gamma}/2\pi$). Here, we experimentally demonstrate that the predicted imprecision is indeed correct. In Fig. S4b a time trace can be seen where the pressure is increased to multiple plateaus (indicated by the right axis in red). The first step in pressure is equal to 2×10^{-3} mbar. By averaging the values of the linewidths measured over 35 s (dashed lines) a distinct increase in the linewidth can be observed, which indicates an experimental imprecision of the pressure of $\sigma_{P,\Gamma,exp} = 2 \times 10^{-3}$ mbar, which is expected for this integration time used.

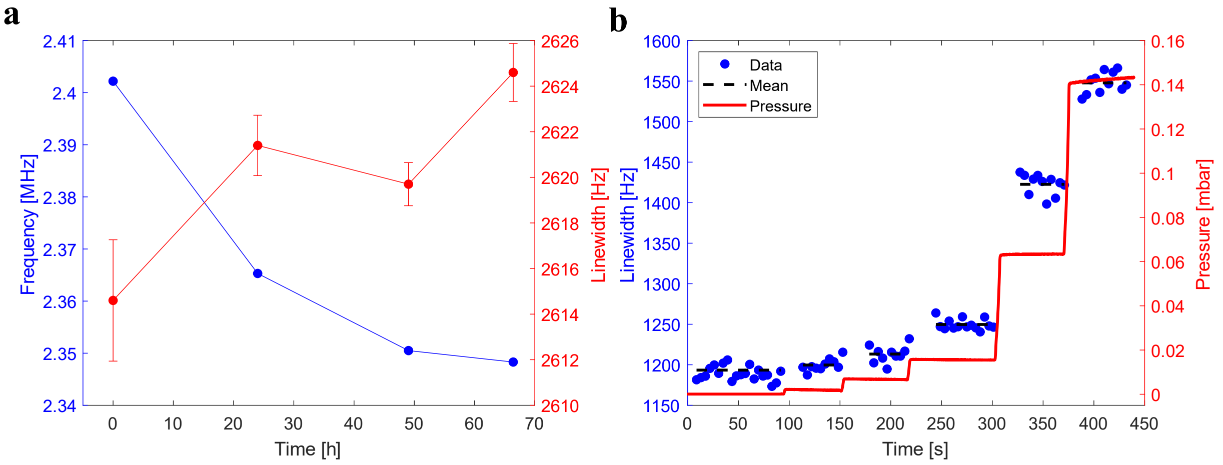

***Fig. S4: a)** Stability of the mechanical frequency and linewidth over 4 days showing a substantial drift and the frequency, but a stable linewidth. The error bars represent the values of the error of the mean. **b)** Linewidth measured as a function of time, while pressure is varied in small steps in the range* $10^{-3} - 10^{-1}$ mbar, *showing a measurable change already at* 2×10^{-3} mbar.
